# Multiple Applications of a Novel Biarsenical Imaging Probe in Fluorescence and PET Imaging of Melanoma

**DOI:** 10.1021/acs.bioconjchem.0c00671

**Published:** 2021-02-12

**Authors:** Mikhail Kondrashov, Samuel P. S. Svensson, Peter Ström, Andreas Westermark, Hanna Jacobson-Ingemyr, Akihiro Takano, Lenke Tari, Miklós Tóth, Minying Cai, Victor J. Hruby, Magnus Schou

**Affiliations:** † Department of Clinical Neuroscience, Center for Psychiatry Research, Karolinska Institutet and Stockholm County Council, 171 77, Stockholm, Sweden; ¶ Biopercept Ltd, PR2 5DB, Barnfield Way, Preston, United Kingdom; # Department of Chemistry, Linkoping University, 581 83, Linkoping, Sweden; ∥ Novandi Chemistry AB, 151 36, Södertälje, Sweden; ‡ Department of Chemistry, University of Arizona, Tucson, Arizona 85721, United States; § AstraZeneca PET Science Centre at Karolinska Institutet, Precision Medicine and Biosamples, Oncology R&D, AstraZeneca, Karolinska Institutet, 17176 Stockholm, Sweden

## Abstract

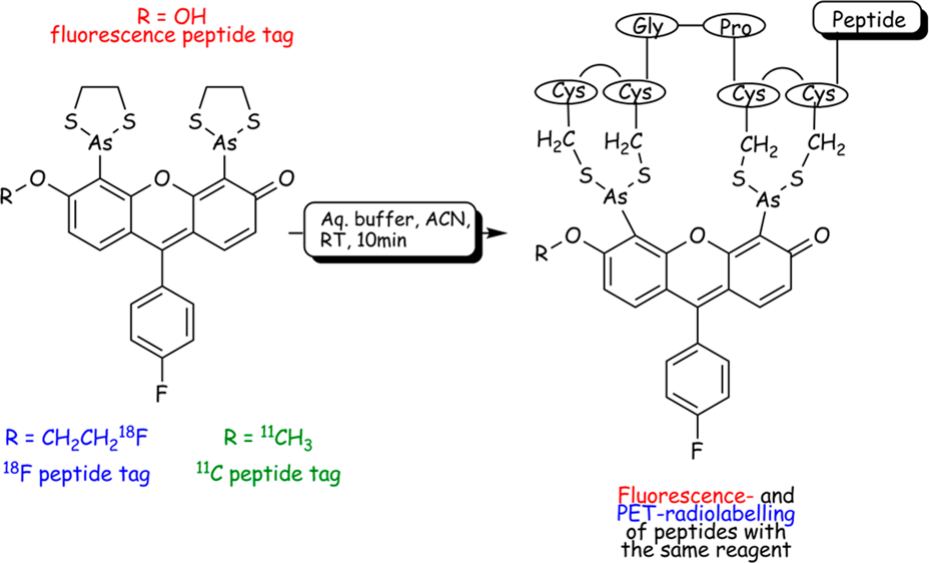

A new fluorescent biarsenical peptide labeling probe was synthesized and labeled with the radioactive isotopes ^11^C and ^18^F. The utility of this probe was demonstrated by installing each of these isotopes into a melanocortin 1 receptor (MC1R) binding peptide, which targets melanoma tumors. Its applicability was further showcased by subsequent *in vitro* imaging in cells as well as *in vivo* imaging in melanoma xenograft mice by fluorescence and positron emission tomography.

Proteins are increasingly important as diagnostic agents and drugs. In 2019, the peptide drug market alone surpassed 70 billion USD,^1^ and a large portion of newly developed positron emission tomography (PET) imaging biomarkers are peptides or antibodies. A fundamental prerequisite for PET imaging is the efficient incorporation of a positron-emitting radionuclide into the target molecule, and it is widely accepted that novel radiolabeling methodologies are required to meet the increasing demand for noninvasive studies of protein distribution *in vivo*. The physical half-life of the radionuclide needs to be compatible with the biological half-life of the imaging probe. Larger proteins, due to their slower biodistribution, require labeling with longer-lived radiometals, whereas smaller peptides or antibody fragments are compatible with labeling using the most used radionuclides in PET, carbon-11 (*t*_1/2_ = 20.4 min) or fluorine-18 (*t*_1/2_ = 109.7 min). The latter has a more convenient half-life, which has sparked the development of numerous methods for its application in peptide labeling. Some notable methodologies include use of [^18^F]SFB,^2^ copper-mediated azide–alkyne cross coupling (AAC),^3^ and more recent metal-free methodologies, such as the strain-promoted AAC^4^ as well as the inverse-electron demand Diels–Alder reaction (Scheme 1a,b).^5^

**Scheme 1:**
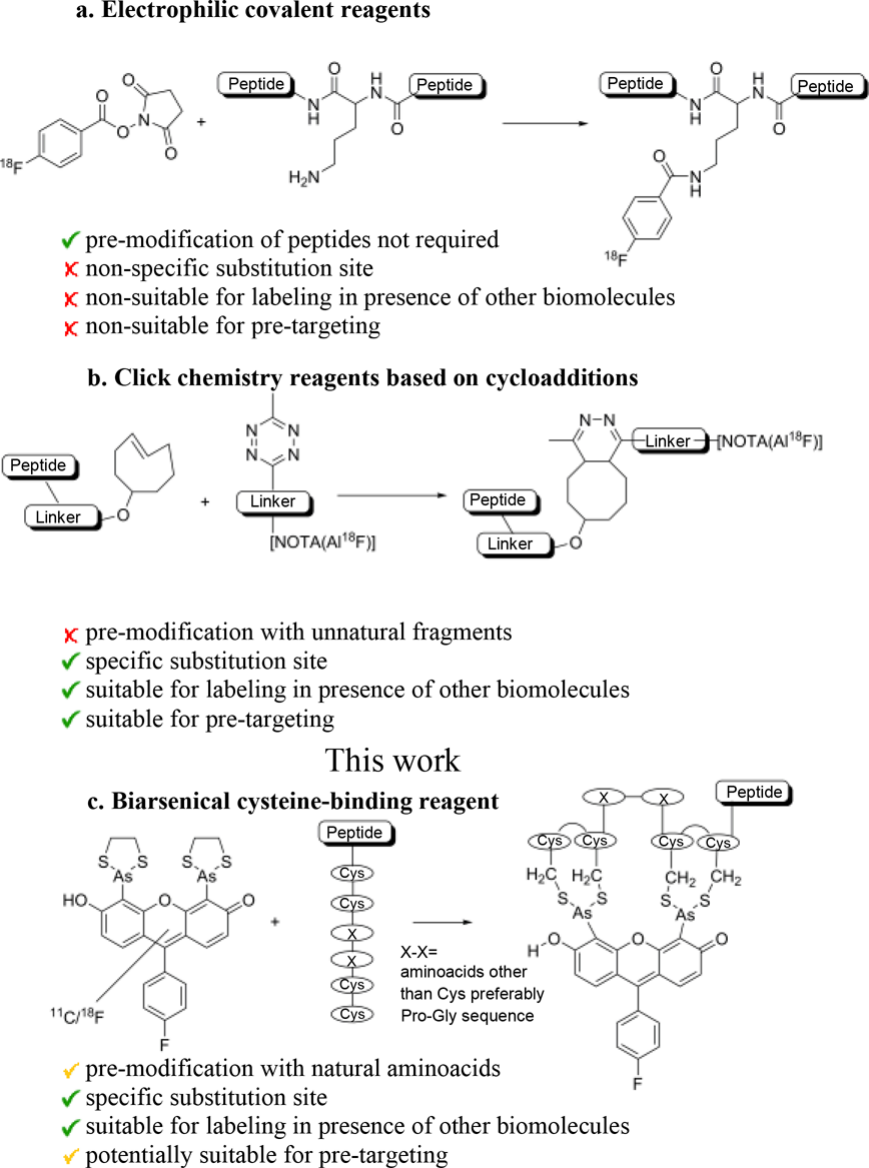
Examples of Peptide Labeling Methodologies: (a) Labeling Lysine Residues with [^18^F]SFB. (b) Inverse-Electron Demand Diels−Alder Coupling. (c) Proposed Modification of FlAsH Fluorescent Labeling of Modified Proteins

In 1998, Griffin et al. described a system for fluorescent imaging using a biarsenical fluorescein analogue system now widely known as FlAsH.^6^ This system relied on a reversible soft Lewis acid–base reaction between As(III) and S(-II) of cysteine. Two arsenic atoms formed four bonds with a Cys-Cys-Pro-Gly-Cys-Cys (CCPGCC) sequence resulting in a high binding constant. Since this sequence is uncommon in natural proteins, it requires installation via genetic alteration of the organism where the protein is expressed; in small peptides, it can be introduced during chemical synthesis. However, as a result, the protein labeling could be performed directly in live cells as it was biocompatible and highly selective. The modification site can be selected in a way in which the overall disturbance of the original protein structure and properties after labeling is minimized. Inspired by this work, we hypothesized that this high-affinity interaction could be exploited for nuclear medicine imaging purposes (Scheme 1c). Importantly, such a concept would allow for validation studies *in vitro* and *in vivo* using an identical imaging probe prior to embarking on the mentioned nuclear medicine imaging.

We herein report the preparation of a novel biarsenical probe, its radiolabeling, and application in the site-specific labeling of peptides. Furthermore, the present study demonstrates the utility of this probe in a variety of studies, including *in vitro* radioligand binding studies in cells and *in vivo* imaging of melanoma tumors in xenograft mice using fluorescence and PET. Molecule **1** was designed with a reactive handle that could be used for introducing both carbon-11 and fluorine-18 via alkylation reactions. The carboxylic acid moiety of the original FlAsH molecule was removed since it was expected to both hamper regioselectivity of the isotopic labeling and the permeability of the labeled probe (Scheme 2).

**Scheme 2:**
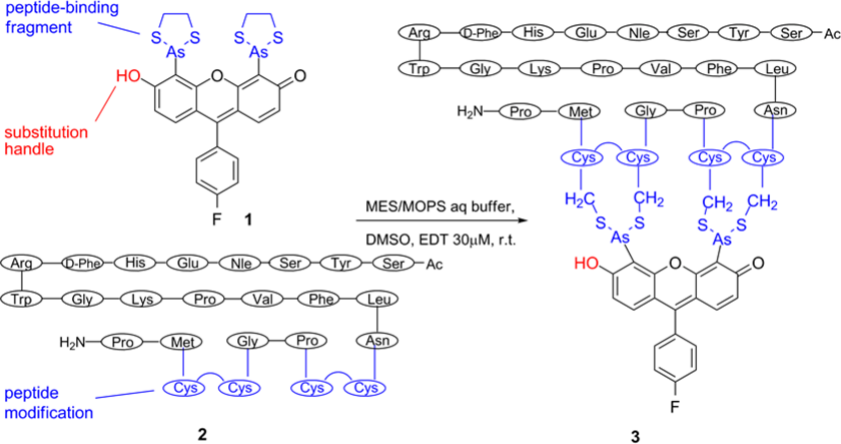
Reaction between NDP-α-MSH and Biarsenical Probe **1**

The MC1R was selected as a target for our investigations. MC1R is overexpressed in melanoma cells^7−12^ and had previously been imaged with positron emission tomography using an ^18^F-labeled α-melanocyte stimulating hormone (MSH) analogue.^11^ To enable labeling with our biarsenical imaging probe, the α-MSH analogue NDP-α-MSH (**2**) with an 11-amino-acid sequence at the C-terminus (containing the CCPGCC fragment) was designed.^11,13^ The peptide was mildly and efficiently labeled with biarsenical **1** to afford the desired biarsenical-MSH peptide **3** (Scheme 2). The introduction of a CCPGCC fragment into a relatively small peptide could have a major effect on its pharmacological properties. The binding affinity of **3** was therefore examined in A375 human melanoma cells. Gratifyingly, the binding of **3** was saturable, of high affinity (3.12 nM), and to a single receptor population with the same Bmax as that observed for MSH which can be exploited in transdermal fluorescence imaging (Figure S6). This fluorescent label demonstrates a broad spectrum of absorption and a high quantum yield which enables detection *in vivo* (Figures S7–8).

Following the injection of **3** (1 μmol) into MC1R tumor-bearing mice, an intense fluorescent staining of the tumor site was observed (Figure 1). Although fluorescent imaging is limited to the tissues that are located proximally to the epidermis, this observation demonstrates the potential utility of **1**.

**Figure 1:**
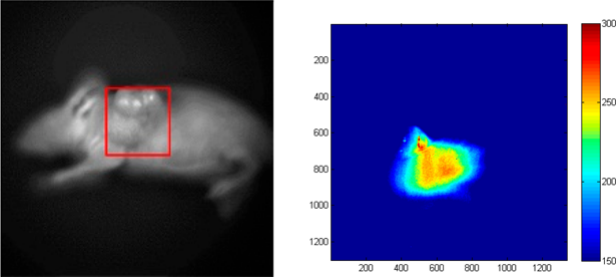
*In vivo* fluorescence labeling of a A375 melanoma xenografted mice 1 h post-injection (right). The tumor site is highlighted by the red box (left).

Encouraged by these promising *in vitro* and *in vivo* results, we investigated the radiolabeling of **1**. A nearly quantitative conversion of [^11^C] methyl triflate into [^11^C]CH_3_-**1** observed after trapping the ^11^C-methylating agent into a solution of the corresponding phenolic precursor and potassium carbonate in acetone (Scheme 3). Two approaches were then evaluated for the subsequent coupling with tetracysteine peptide **2**. The first one included isolation of [^11^C]CH_3_-**1** using semipreparative HPLC followed by its coupling with **2** in a mixture of acetonitrile and aqueous buffer. To our delight, a 5 min incubation at room temperature furnished the [^11^C]**3** peptide in >90% radiochemical purity. The concentrated aqueous solution of [^11^C]CH_3_-**1** underwent substantial chemical decomposition due to the intense β+- and γ-radiation (so-called radiolysis). Fortunately, it could be prevented by addition of a sodium ascorbate—a typical antioxidant reagent used in such cases. In the second protocol, crude [^11^C]CH_3_-**1** was instead directly incubated with **2** and [^11^C]**3** was isolated using HPLC. The radiochemical purity of the reaction mixture was about 80%, and the amount of peptide had to be increased from 0.1 mg to 1.5–2.5 mg because of the competition for the tetracysteine motif between the biarsenic moieties in [^11^C]CH_3_-**1** and its unlabeled phenolic precursor. In addition, HPLC isolation required the use of two mobile phases and resulted in a low overall radiochemical yield of [^11^C]**3** (2% based of starting [^11^C]CH_4_, decay-corrected). Nevertheless, [^11^C]**3** could be obtained in >95% radiochemical purity and >300 GBq/μmol molar activity and was radiochemically stable in solution for at least 1 h, thus allowing its application in proof-of-concept PET imaging studies in mice. [^11^C]**3** (10–15 MBq) was injected intravenously into tumor-bearing mice (*n* = 4). Two syngeneic tumor models were used for this purpose, B16/F10 and A375.^11^ Following intravenous injection of [^11^C]**3**, the highest radioactivity was found in the gut region (liver, intestines, etc.), whereas the brain was almost devoid of radioactivity (Figure 2a). Consistent with the findings of Cheng et al.,^11^ no obvious retention of radioactivity was observed in tumors originating from the A375 cell line (Figure 2b), in which the MC1R expression is substantially lower. In contrast, the tumor was clearly visible following injection of [^11^C]**3** peptide in B16/F10 mice, which indicates that [^11^C]**3** binds MC1R *in vivo*. The tumor to muscle ratio for both cell lines was lower than in the study by Cheng, which indicates that this peptide conjugate may be inferior as a PET-tracer; however, a head-to-head comparison of the two tracers should be performed in order to shed further light on this matter.

**Scheme 3:**
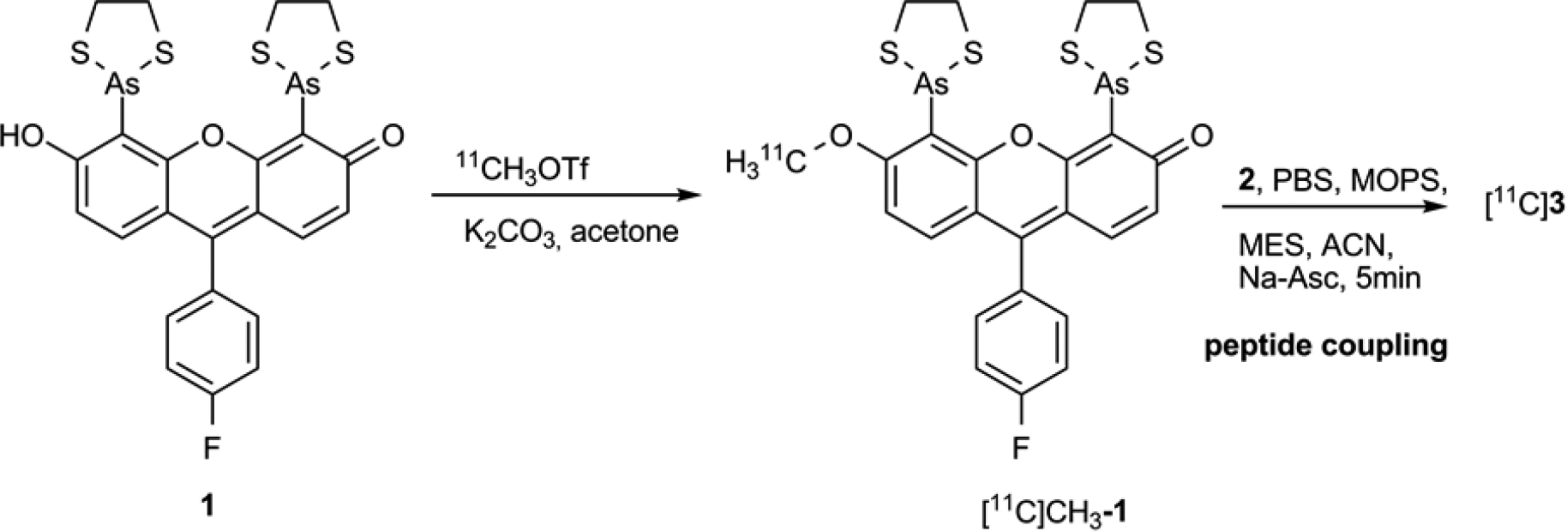
Carbon-11 Labeling of **1**, Followed by the Coupling to the Peptide

**Figure 2:**
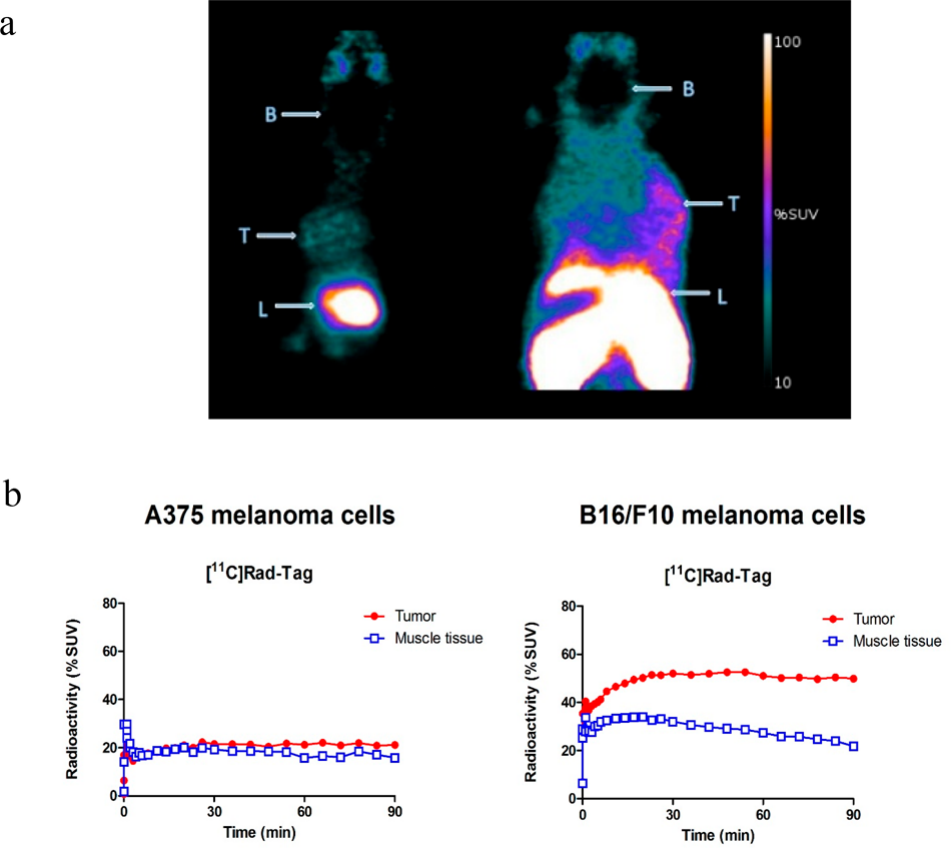
(a) Color-coded summation PET images (5–60 min) showing the distribution of [^11^C]**3** in a A375 (left) and B16/F10 (right) mice. Apparent difference in uptake in the liver is connected to the different horizontal planes of the scan, due to the normal variation of tumor growth and animal positioning in the scanner. Abbreviations: B, brain; T, Tumor; L, Liver. (b) Time–radioactivity curves in tumor-bearing mice following IV injection of [^11^C]**3**.

Larger proteins such as antibodies typically have longer biological half-lives (from days to weeks), which is incompatible with short-lived radionuclides, such as ^11^C and ^18^F. Long-lived radiometals, e.g., ^89^Zr (*t*_1/2_ = 78.4 h), are therefore typically preferred for this purpose. However, the substantial radiation burden associated with the longer half-life has called for pretargeting approaches, in which the large molecule of interest is preadministered into a patient or a research subject. Then, PET imaging is conducted at a separate time point (or multiple time points) after the initial molecule has been cleared from plasma, and the bioconjugation takes place *in vivo* with probes labeled using a short-lived radionuclide. The most notable reaction for this purpose is the inverse-electron demand Diels–Alder reaction, which relies on the introduction of a trans-cyclooctene moiety into the protein of interest.^5^ FlAsH labeling of genetically modified proteins was performed directly in living cells.^6,14,15^ Thus, it is possible to foresee a pretargeting labeling approach for **1**. Although very appealing, this approach is associated with some challenges including the potential *in vivo* oxidation of the tetracysteine motif as well as the nonspecific binding of biarsenicals to cysteine residues which was observed in cell imaging.

Among several criteria important for the success of a pretargeting approach, the probe should first exhibit reasonable plasma stability *in vivo* to allow for its interaction with the target protein during the time frame of the PET study. Second, high background affinity to any organ would severely hamper protein imaging in that organ. In order to test if **1** fulfilled these prerequisites, a two-hour dynamic PET measurement was performed after an intravenous injection of [^11^C]CH_3_-**1**, with the upper body of a nonhuman primate subject (NHP) in the field of view. The highest radioactivity following intravenous injection of [^11^C]CH_3_-**1** was observed in the liver, followed by the heart. The brain was almost devoid of radioactivity, indicating that the compound does not pass the blood-brain barrier (Figure 3a). The high radioactivity in the liver and the low uptake in the brain likely prohibits the use of **1** for pretargeting in these organs. On the other hand, while the intermediate background radioactivity surely hampers sensitivity, it cannot be ruled out that lungs and heart can be targeted using **1**, assuming that the target protein density is sufficiently high. Blood samples were taken at multiple time points during the NHP PET measurements for the purpose of analyzing radiolabeled metabolites in plasma. The recovery of radioactivity after separation of the blood cells and plasma proteins was moderate (the former dropped from 89% to 7% during the 2 h scan time, the latter, from 80% to 60%), indicating that a substantial amount of the compound was in the bound state. However, more than 60% of the radioactivity in purified plasma was constituted by the parent compound at 120 min after injection, thus demonstrating that the plasma stability of [^11^C]CH_3_-**1** could be satisfactory for a pretargeting approach to PET imaging (Figure 3b).

**Figure 3:**
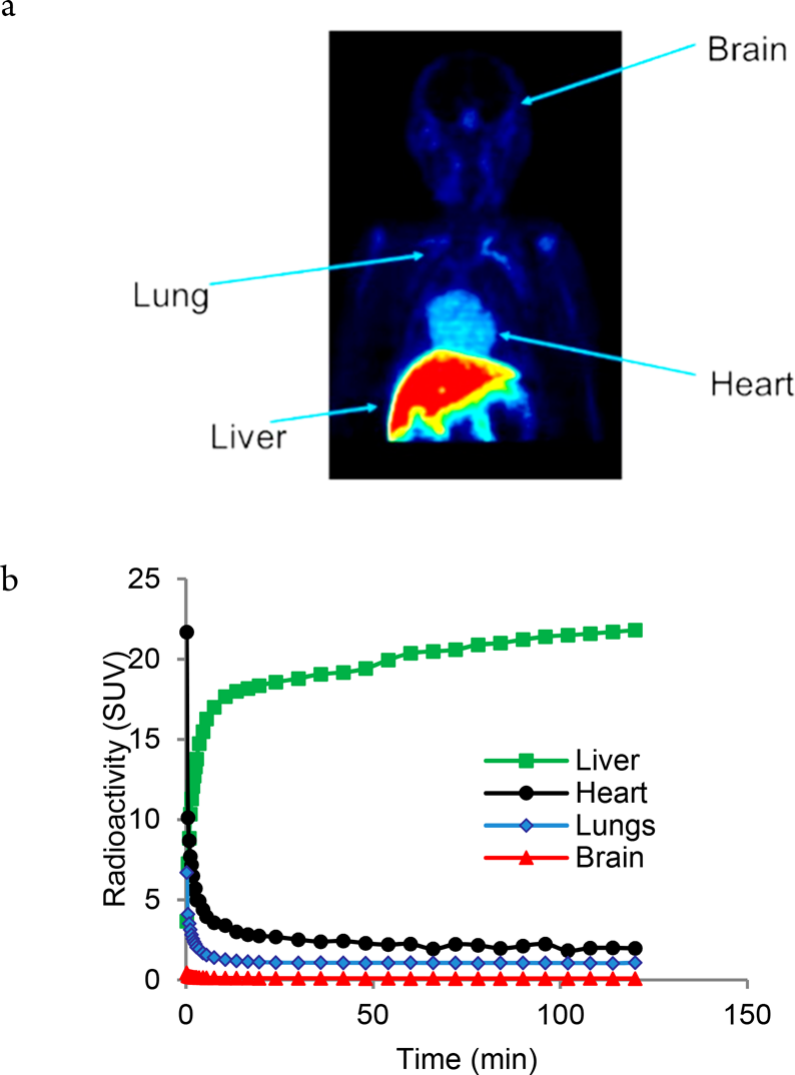
(a) Summation PET image of NHP showing the distribution of [^11^C]CH_3_-**1** from 3 to 60 min after intravenous injection. (b) Time–activity curves showing organ distribution following intravenous injection of [^11^C]CH_3_-**1**.

Fluorine-18 is the most widely used radionuclide in PET. It has several advantages, including its convenient half-life and facile large-scale production, which allow for centralized preparation and shipment of ^18^F-labeled tracers to distant sites that lack an on-site cyclotron. We selected fluoroethylation as a method of labeling with fluorine-18. Though preliminary attempts at alkylating **1** using 1-bromo-2-[^18^F]fluoroethane were unsuccessful, 2-[^18^F]fluoro-ethyl triflate^16^ was found effective as an electrophile and led to the formation of [^18^F]F-C_2_H_4_-**1** in >60% radiochemical yield. The product was readily purified by semipreparative HPLC, resulting in a fraction of >95% radiochemical purity and specific activity of >160GBq/μmol. As in the case of [^11^C]CH_3_-**1**, [^18^F]F-C_2_H_4_-**1** underwent radiolysis and required stabilization with sodium ascorbate. In this context, it is worth noting that we were not able to isolate [^11^C]CH_3_-**1** or [^18^F]F-C_2_H_4_-**1** via solid-phase extraction (SPE) in good radiochemical yield. Probable reasons for this were decomposition via radiolysis and low recovery from the SPE due to the lipophilic nature of the biarsenicals. Nevertheless, the product solution obtained after HPLC was found suitable for coupling with **2** according to a similar procedure as that used in the ^11^C-labeling. Following a detailed investigation of the reaction, it was found that the inclusion of dithiothreitol (DTT) was beneficial for the coupling reaction (Scheme 4). We hypothesize that the action of DTT is twofold. First, it acts as a reductant of disulfide bonds in the CCPGCC fragment, which could hamper reactivity toward the arsines. Second, DTT catalyzes the As–S bond cleavage reaction. The longer half-life of ^18^F also allows further operations to be performed with the product; hence, the labeled peptide could be separated from the excess of the unlabeled peptide and other reaction components by a preparative HPLC and isolated in ∼1 GBq yield.

**Scheme 4:**
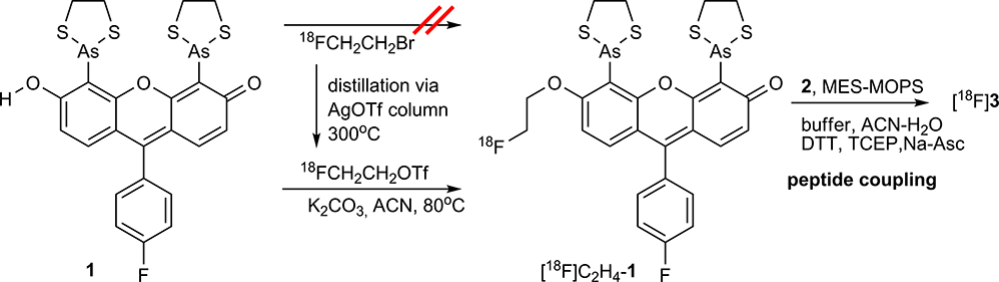
Fluorine-18 Labeling of **1**, Followed by the Coupling to the Peptide

In summary, we herein report the first successful multiple applications of the biarsenical probe **1** in the rapid multimodal (fluorescence- and radio-) labeling of peptides for *in vivo* and *in vitro* molecular imaging. An advantage of the current methodology is that it requires proteins to be modified with a fragment consisting of only natural amino acids, that can be conveniently installed using automated synthesizers or directly expressed in a genetically altered living organism. It can serve as a valuable addition to the library of existing methods of protein labeling.

## Supporting Information Available

Description of synthesis of precursors and references, procedures of radiosynthesis, general data regarding the animal studies (PDF)
