# Supplementary material for: Multiple Applications of a Novel Biarsenical Imaging Probe in Fluorescence and PET Imaging of Melanoma
Source: Bioconjug Chem. 2021 Feb 12;32(3):497–501. doi: 10.1021/acs.bioconjchem.0c00671 (PMC8023571; doi:10.1021/acs.bioconjchem.0c00671)
Supplement: Supplementary file 1 — bc0c00671_si_001.pdf [file bc0c00671_si_001.pdf]

Supplementary Information

Multiple Applications of a Novel Biarsenical Imaging Probe in Fluorescence and PET Imaging of Melanoma

Mikhail Kondrashov,[†] Samuel P.S. Svensson,[¶#] Peter Ström,[1] Andreas Westermark,[†] Hanna Jacobson-Ingemyr,[†] Akihiro Takano,[†] Lenke Tari,[†] Miklós Tóth,[†] Minying Cai,[‡] Victor J. Hruby,[‡] and Magnus Schou.[†§]

† Department of Clinical Neuroscience, Center for Psychiatry Research, Karolinska Institutet and Stockholm County Council, 171 77, Stockholm, Sweden.

¶ Biopercept Ltd, PR2 5DB, Barnfield Way, Preston, United Kingdom

Dept of Chemistry, Linkoping University, 581 83, Linkoping, Sweden

1 Novandi Chemistry AB, 151 36, Södertälje, Sweden

‡ Department of Chemistry, University of Arizona, PO Box 210041, USA

§ AstraZeneca PET Science Centre at Karolinska Institutet, Precision Medicine and Biosamples, Oncology R&D, AstraZeneca, Karolinska Institutet, 17176 Stockholm, Sweden

Contents

General 1

Synthesis of precursor 1 2

Synthesis of references 4

General radiochemistry 5

Radiochemical syntheses 5

PET-Imaging with mice 9

PET imaging in a non-human primate 10

Binding assay data 11

References 12

General

All chemicals and solvents were obtained from Sigma-Aldrich (Sweden) and used without further purification, unless specified otherwise. Anhydrous acetonitrile was purchased from Acros**.** Peptide **2** was provided by RedGlead Discovery AB. All reactions sensitive to moisture or oxygen were carried out under Ar atmosphere in flame-dried glassware. THF was distilled over potassium and CH_2Cl_2 was distilled over CaH_2 prior to use. Full assignment of ^1H and ^{13}C chemical shifts are based on the 1D and

2D FT NMR spectra on a Bruker Avance III 400 MHz instrument. Solvent peaks in ^{13}C- and ^{1}H-NMR were used as chemical shift references.

High performance liquid chromatography analysis (HPLC) was performed using a Hitachi L-6200 gradient pump and a Hitachi L-4000 variable wavelength UV-detector in a series with a Bioscan β+-flow detector. The products containing the xanthenone core of compound **1** could be conveniently observed at a wavelength of 380 or 465nm. Analytical HPLC analysis was performed using a reverse phase column (XBridge, C18, 5 µm, 4.6 x150 mm), unless otherwise noted.

Synthesis of precursor 1

2,5-di-(1,3,2-dithiarsolan-2-yl)-9-(4-fluorophenyl)-6-hydroxy-3-fluorone (**1**) was synthesised similarly to a published procedure[1] in following steps.

Bis(2,4-dimethoxyphenyl)-4-fluorophenylmethanol

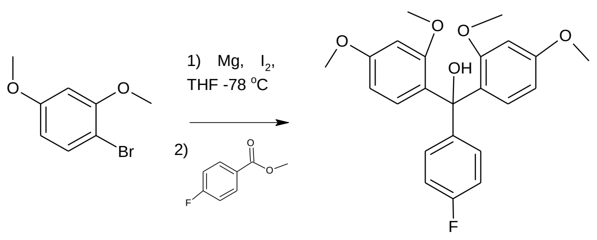

Magnesium turnings (6.923 g, 285 mmol) and a few crystals of I_2 are placed in a 1-L three-neck round-bottom flask fitted with a dropping funnel and a condenser. A solution of 2,4-dimethoxybromobenzene (42.7 mL, 294 mmol) in 60 mL of anhydrous THF is added dropwise to the magnesium. Additional THF (~300 mL) is added by portion to cool the reaction. The mixture is stirred for 30 min. The resulting Grignard reagent (2,4-dimethoxyphenylmagnesium bromide) is cooled in a dry ice/acetone bath before a solution of 4-fluoro methyl benzoate (15,5 mL, 120 mmol) in 110 mL of dry THF is added dropwise. The mixture is stirred overnight and then quenched with 1 L of distilled water and neutralised with 2 N HCl. The resulting green mixture is extracted with ethyl acetate (3x300 mL). The combined extracts are dried over $MgSO_4$, filtered, and evaporated to dryness. The residue is purified by precipitation using EtOAc and heptane to afford an off-white solid (37 g, 77%).
^{1}H NMR (400 MHz, $CDCl_3$) δ 7.27 – 7.18 (m, *J* = 8.4, 5.7 Hz, 2H), 7.00 – 6.81 (m, 4H), 6.50 (d, *J* = 2.0 Hz, 2H), 6.40 (dd, *J* = 8.6, 2.1 Hz, 2H), 5.12 (s, 1H), 3.80 (s, 6H), 3.54 (s, 6H). ^{13}C NMR (101 MHz, $CDCl_3$) δ 161.64 (d, *J* = 244.2 Hz), 143.29 (d, *J* = 3.2 Hz), 129.45 (d, *J* = 7.9 Hz), 113.86 (d, *J* = 21.1 Hz).

9-(4-fluorophenyl)-6-hydroxy-3-fluorone

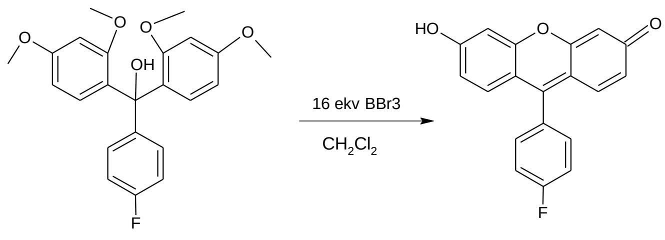

A solution of bis(2,4-dimethoxyphenyl)-4-fluorophenylmethanol (17 g, 42,7 mmol) in 500 mL of dry CH_2Cl_2 is cooled to -78 °C using a dry ice/EtOH bath before BBr_3 (66 mL, 683 mmol) is added over 40 minutes. The mixture is allowed to warm to room temperature gradually and stirred overnight. The

reaction mixture is transferred into a 2 L of cold water using cannula. The layers were separated, and water was extracted with CH_2Cl_2 (2x400 mL) and EtOAc (5x400 mL). The red precipitate was filtered off and discarded. The combined extracts were dried over Na_2SO_4 and filtered and concentrated. The residue is purified by precipitation using methanol and EtOAc to afford mustard-coloured powder (2.38 g, 18 %).
^{1}H NMR (400 MHz, MeOD) δ 7.81 (d, *J* = 9.3 Hz, 2H), 7.69 – 7.64 (m, 2H), 7.55 – 7.48 (m, 2H), 7.39 (d, *J* = 2.3 Hz, 2H), 7.30 (dd, *J* = 9.3, 2.3 Hz, 2H).

9-(4-fluorophenyl)-6-hydroxy-3-fluorone-2,5-dimercuric trifluoroacetate

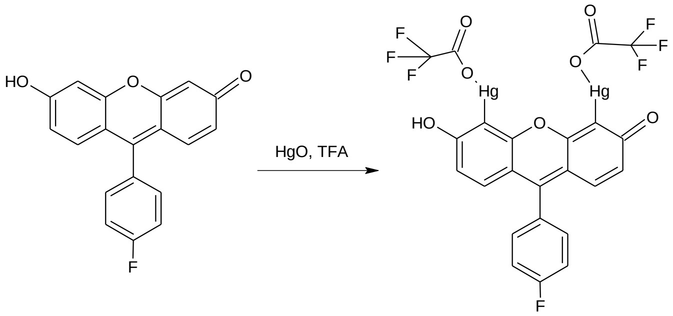

HgO (2.77 g, 12.8 mmol) was dissolved in 30 mL of trifluoroacetic acid, 9-(4-fluorophenyl)-6-hydroxy-3-fluorone (1.95 g, 6.39 mmol) was added and the mixture was stirred overnight. The TFA was evaporated and 50 mL of water was added. The product was filtered off and washed with 3 portions of water. The product was dried using high vacuum pump to yield orange solid (5.06 g, 85%).
^{1}H NMR (400 MHz, DMSO+TFA) δ 7.74 – 7.66 (m, 2H), 7.61 – 7.54 (m, 4H), 7.31 (d, *J* = 9.1 Hz, 2H).

2,5-di-(1,3,2-dithiarsolan-2-yl)-9-(4-fluorophenyl)-6-hydroxy-3-fluorone (1)

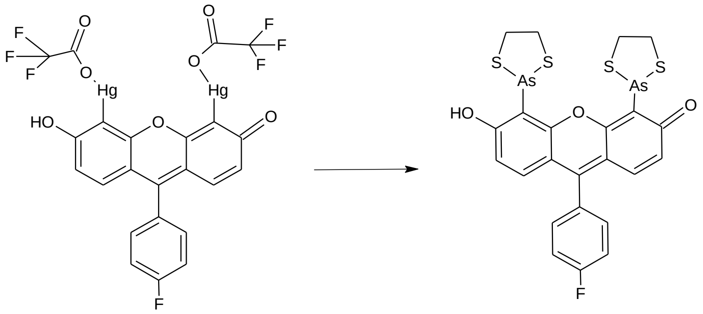

The 9-(4-fluorophenyl)-6-hydroxy-3-fluorone-2,5-dimercuric trifluoroacetate (5.0 g, 5.37 mmol) was dissolved in 53 mL of dry N-methylpyrrolidone under argon atmosphere, $AsCl_3$ (9.0 mL, 107 mmol) and N,N-diisopropylethylamine (7.5 mL, 43.0 mmol) were added via syringe. 32 mg of palladium (II) acetate was added and the mixture was stirred at 55 °C for two hours and then at room temperature overnight. The reaction mixture was transferred into a 500-mL Erlenmeyer flask containing well-stirred 320 mL of 1:1 vol/vol 0.3 M potassium phosphate buffer at pH 6.9 and acetone. 21 mL of 1,2-ethanedithiol was added to the solution and stirred for 30 minutes, and then 100 mL of chloroform was added and stirred for additional 20 minutes. The organic phase was separated, and the water was extracted 3 times with chloroform, the combined organic layers were washed with water and dried over Na_2SO_4, filtered and concentrated. Methanol was added, large amount of white precipitate was formed, also a small amount of dark red liquid at the bottom of the flask. The precipitate was

decanted, the remaining red liquid was dissolved in chloroform and collected. This procedure was repeated three times. The combined crude product was concentrated and purified by column chromatography using neat toluene to toluene:acetone 50:4 to yield a orange-red solid (1.0 g, 31%). ^{1}H NMR (400 MHz, $CDCl_3$) δ 10.57 (s, 1H), 7.41 – 7.21 (m, 2H), 7.08 – 6.97 (m, 1H), 6.62 (broad s, 1H), 3.88 – 3.27 (broad m, 1H).

Synthesis of references

Synthesis of [^{12}C]CH_3-1

To a suspension of **1** (27 mg, 42 µmol) and potassium carbonate (11mg, 84µmol, 2 eq) in 3 mL of dry acetone, methyl nosylate (9.1mg, 42µmol, 1 eq) was added and the mixture was heated to 60°C under microwave irradiation conditions for 60 min. The solvent was evaporated, and the residue was purified by HPLC (C8) column chromatography using 75% acetonitrile in 25 mM ammonium acetate at 2.0 mL/min. The resulting HPLC fraction was evaporated by flushing with a stream of nitrogen, to produce 11mg of brown solid, which was re-purified on silica using toluene:ethyl acetate (10:1). The isolated fraction was evaporated to produce 4.5mg (16%) of [^{12}C]CH_3-**1** as a red powder, which was pure according to LC-MS (m/z 653 $[M+H]^+$).

Synthesis of [^{19}F]F-C_2H_4-1

To a suspension of **1** (30 mg, 48 µmol) and potassium carbonate (13.2mg, 96µmol, 2 eq) in 3 mL of dry acetone, 2-fluoro-ethyl tosylate (21mg, 96µmol, 2 eq) was added and the mixture was heated to 120°C under microwave irradiation conditions for 10 min. The solvent was evaporated and the residue was purified by column chromatography on silica using toluene:ethyl acetate (10:1). The isolated fraction was evaporated to result in 10mg of red powder. The product could be re-purified on C8 HPLC column with ACN-NH_4OAc(25mM in water) 70:30. The isolated fraction was evaporated by flushing with a stream of nitrogen, redissolved in acetonitrile and evaporated again. [^{19}F]F-C_2H_4-**1** was obtained as a red powder (8mg, 24%) and was pure according to LC-MS (m/z 685 $[M+H]^+$).

Synthesis of [^{12}C]3

The solution of [^{12}C]CH_3-**1** (1.5mg in 500µL of DMSO) was added to a mixture of peptide **2** (6.0mg in 250µL of 0.1%TFA), ethanedithiol (10µL, 10mM in DMSO), ACN (250µL), MES-Na salt (50µL, 1.0M aq) and MOPS-Na salt (500µL, 100mM aq), the mixture was kept at room temperature for 10 minutes. The reaction mixture was injected on a preparative short C18 column, which was eluted at a flow 5.0mL/min with a mixture of ACN-NH_4OAc(50mM in water) 45:55. The first major peak observed at 465nm was collected. Isolated fraction was concentrated in a stream of nitrogen and reinjected on Kromasil C18, 7µm 250x10mm with the same eluent, flow 2.0mL/min. Isolated fraction was evaporated by flushing with a stream of nitrogen, redissolved in acetonitrile and evaporated again. This resulted in 3.5mg (50%) of the product. MS calculated $[M+2H]^{2+}$ 1640.67, observed 1640.58.

Synthesis of [^{19}F]3

The solution of **[^{19}F]F-C_2H_4-1** (10µL, 10µg/µL in DMSO) was added to a mixture of peptide 2 (10µL, 10µg/µL in 10µL of 0.1%TFA), ACN (20µL), DTT (2µL 0.2M solution), MES-Na salt (10µL, 300mM) and MOPS-Na salt (10µL, 300mM), the mixture was kept at room temperature for 10 minutes. The reaction mixture was injected on a preparative ACE 5 C18-HL (250x10mm) column, which was eluted at a flow 5.0mL/min with solvents A (0.085% TFA in ACN) and B (0.1% TFA in water) using a following gradient: 0 to 2min 10% A, 2 to 10min gradient 10% to 90% A, 10 to 13 min 90% A. The first major peak observed

at 465nm was collected. Isolated fraction could be used directly as a reference solution. MS $[M-H]^+$ 3311.40 calculated, observed 3311.12.

General radiochemistry

All experiments were performed in accordance with local and national rules and laws that govern the work with open sources of radiation. Radioisotopes were produced on a GEMS PETtrace cyclotron (General Electrics Medical Systems, Uppsala, Sweden). No-carrier-added $[^{11}C]CH_4$ was produced using 16.5 MeV protons in the $^{14}N(p,a)^{11}C$ nuclear reaction on a mixture of nitrogen and hydrogen gas (10% hydrogen). $[^{11}C]CH_4$ was converted to $[^{11}C]CH_3I$ by radical iodination in a gas-phase recirculation system and swept in a stream of helium through a heated glass column containing silver triflate impregnated on graph-pac to produce $[^{11}C]CH_3OTf$. $[^{18}F]$-fluoroethyl triflate was produced according to a published procedure.[2] The identities of the radiolabelled products were established by coelution with non-radiolabelled reference standards. All reported yields are non-decay corrected, unless noted otherwise. The syntheses times are calculated from the moment of starting the delivery of radionuclides from the cyclotron to synthetic modules.

Radiochemical syntheses

Preparation of $[^{11}C]CH_3$-1.

$[^{11}C]CH_3OTf$ was trapped in a solution of **1** (0.3-0.7mg) in acetone (400µL) containing potassium carbonate (1.5-2.5mg) at r.t. After one minute the reaction mixture was diluted with water (0.6mL) and injected on a preparative ACE 5 C18-HL (250x10mm) column. The column was eluted with EtOH-AmF(0.1M) 40:60 mixture containing 500mg/L of sodium ascorbate at a flow 3.0mL/min for 10 min followed by EtOH-AmF(0.1M) 80:20 mixture containing 500mg/L of sodium ascorbate at a flow of 3.5mL/min. The product fraction was collected and immediately evaporated in a coil evaporator, which was rinsed by 6mL of PBS or 6mL of PBS-propylene glycol-ethanol solution (90:7:3) with 60mg of sodium ascorbate resulting in a >95% RCP product. RCY 1.4% (based on the delivered $[^{11}C]CH_4$). Total synthesis time 40min.

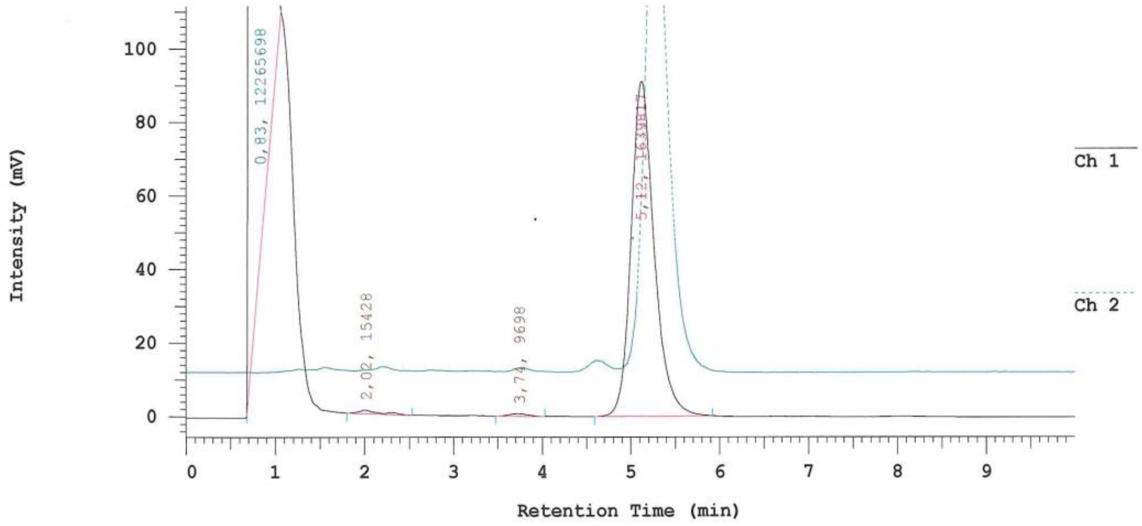

No.	RT	Area	Conc 1	BC
1	1,57	24391	0,941	MC
2	2,21	9416	0,363	MC
3	3,79	12175	0,470	MC
4	4,64	44727	1,725	MC
5	5,27	2501465	96,501	MC
		2592174	100,000	

Figure S1. Typical HPLC chromatogram of the isolated product [^{11}C]CH$_3$-**1** coinjected with reference [^{12}C]CH$_3$-**1**. Blue trace presents UV (254nm), black trace – radiodetector. Column Ascentis RP-Amide. Eluent ACN:AmF (0.1M) 65:35. Flow 2.5mL/min.

Synthesis of [^{11}C]3. Method A.

The [[^{11}C]CH$_3$-**1** was produced as described before, rinsed off the evaporator by pure PBS and added into a vial containing a mixture of peptide **2** (0.1-0.3mg in 125µL of 0.1%TFA), ACN (125µL), EDT (10µL, 10mM solution in DMSO), MES-Na salt (100µL, 1.0M) and MOPS-Na salt (1 mL, 100mM). The reaction was kept at r.t. for 5min then diluted with water and passed through SPE cartridge (HLB, 1cc). The cartridge was rinsed with H_2O (5mL) and eluted with EtOH (200µL) followed by PBS (2mL). RCY 0.35% (based on the delivered [^{11}C]CH$_4$). Total synthesis time 50min.

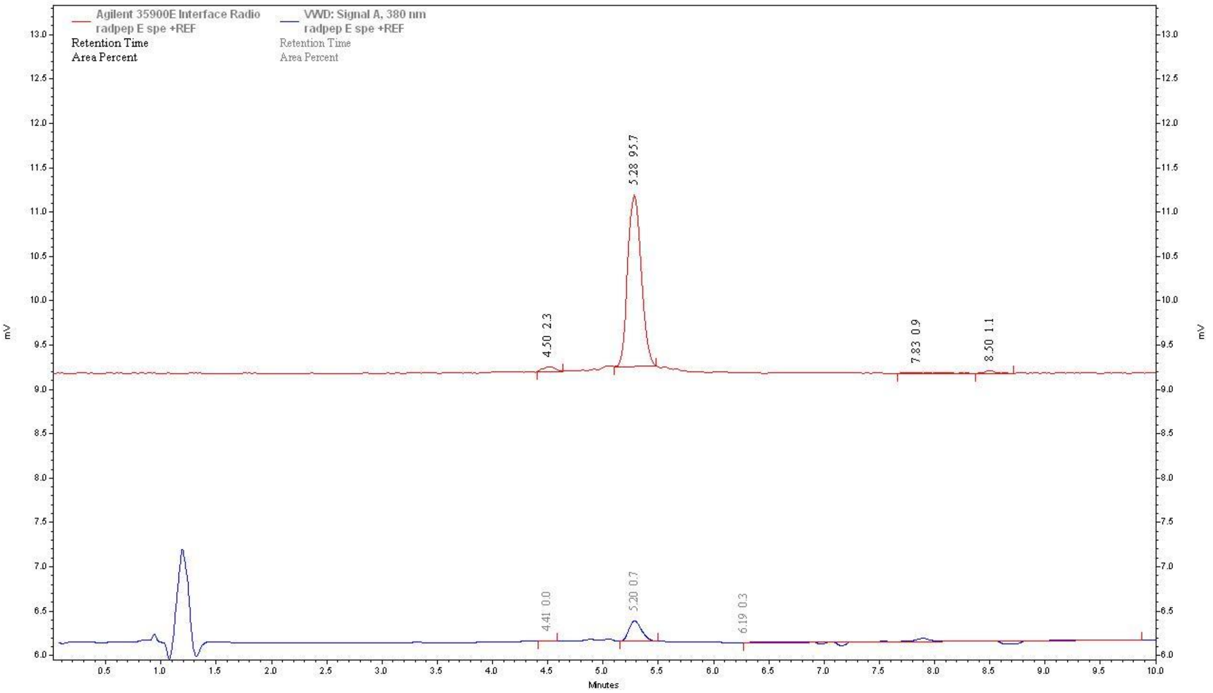

Figure S2. Typical HPLC chromatogram of the isolated product [^{11}C]**3** coinjected with reference [^{12}C]**3**. Blue trace presents UV (380nm), red trace – radiodetector. Column C18 µBondapak analytical. Eluent ACN:AmF (0.1M) 20:80 gradient to ACN:AmF (0.1M) 80:20 in 10min. Flow 3.0mL/min.

Synthesis of 3. Method B.

[[^{11}C]CH$_3$-OTf was trapped in a solution of **1** (0.3-0.7mg) in acetone (400µL) containing potassium carbonate (1.5-2.5mg) at r.t. After one minute, a mixture containing peptide **2** (4.4mg in 100µL of 0.1%TFA), ACN (100µL), EDT (10µL, 10mM solution in DMSO), MES-Na salt (100µL, 1.0M) and MOPS-Na salt (1 mL, 100mM) was added. After 5 min the reaction mixture was diluted with water (1mL) and injected on a preparative ACE 5 C18-HL (250x10mm) column. The column was eluted at a flow of 6.0mL/min with water for 2 min, followed ACN-AmF(0.1M) 35:65 mixture for 10 min followed by ACN-AmF(0.1M) 40:60 mixture. The product fraction was collected, diluted with water (50mL), passed through an SPE cartridge (HLB, 1cc) The cartridge was rinsed with H_2O (5mL) and eluted with EtOH (200µL) followed by PBS solution (2mL). RCY 0.33% (based on the delivered [^{11}C]CH$_4$). Total synthesis time 60min.

Preparation of [^{18}F]F-C_2H_4-1.

[^{18}F]-fluoroethyl triflate was synthesized according to a published procedure[2] and trapped in a mixture of **1** (0.3-0.5mg) and anhydrous potassium carbonate (3-5mg) in 400µL of anhydrous acetonitrile at room temperature. The mixture was then heated at 80°C for 5 minutes, cooled to 40°C, diluted with a solution of 15mg of sodium ascorbate in 0.6mL of acetonitrile and 1.0mL of water and injected on a preparative ACE 5 C18-HL (250x10mm) column. The column was eluted with ACN-H_2O 75-25 mixture, containing 4g/L of sodium ascorbate at a flow of 7.0mL/min. The product was collected at 6-7min in 40% RCY (based on the total activity distilled to the reactor during [^{18}F]-fluoroethyl triflate production). Total synthesis time 60min.

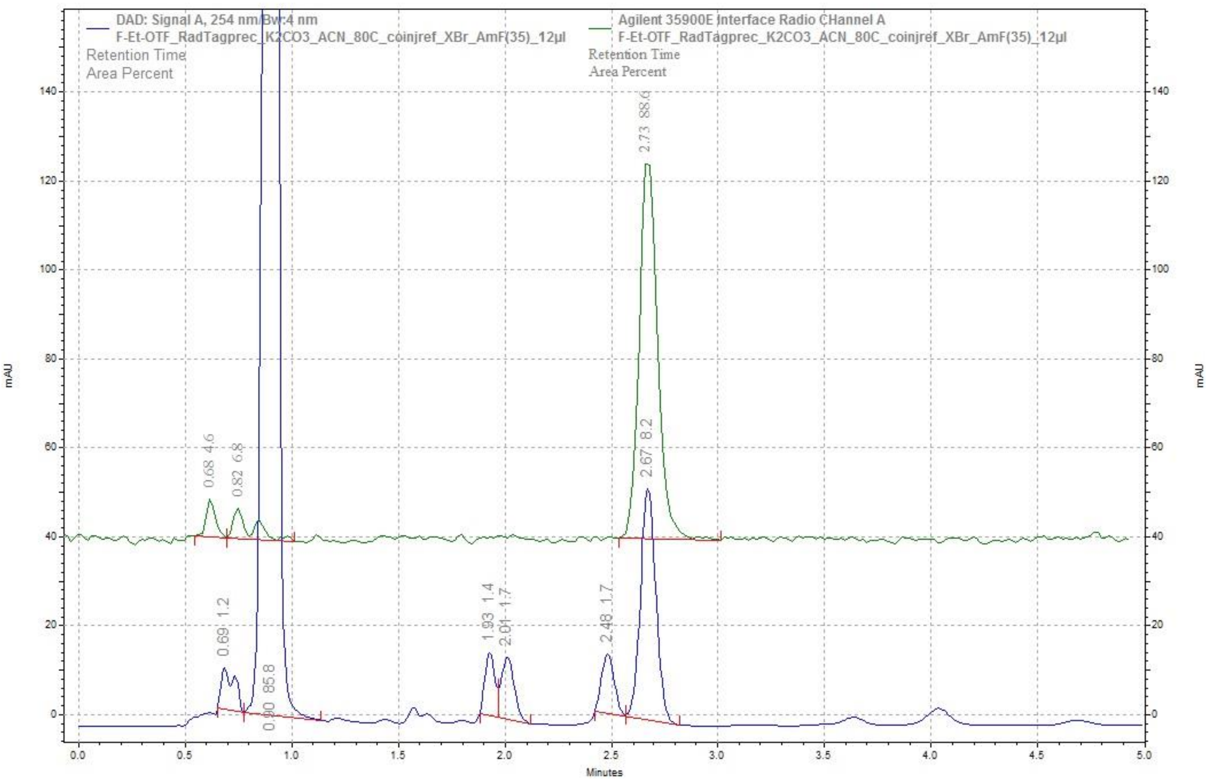

Figure S3. Typical HPLC chromatogram of the crude reaction mixture [^{18}F]F-C_2H_4-**1** coinjected with reference [^{19}F]F-C_2H_4-**1**. Blue trace presents UV (254nm), green trace – radiodetector. Eluent ACN:AmF (0.1M) 65:35. Flow 2.5mL/min.

Synthesis of [^{18}F]3.

The isolated HPLC fraction of [^{18}F]F-C_2H_4-**1** was added to a mixture of peptide **2** (0.58mg in 50µL of 0.1%TFA), ACN (50µL), TCEP (6µL of 0.2M solution), DTT (80µL 0.2M solution), MES-Na salt (100µL, 300mM) and MOPS-Na salt (100µL, 300mM), which was prepared at least 30min in advance. After that, the mixture was kept at r.t. for 10min, diluted with H_2O (80mL) and ammonium phosphate buffer (pH 2.2, 0.2M, 5mL) and passed through SPE cartridge (tC18, 1cc, 50mg). The cartridge was rinsed with H_2O (5mL) and eluted with EtOH (600µL) followed by H_2O (1.4mL). Resulting eluate was analysed to confirm RCP of ~90%, RCY 26% (based on the total activity distilled to the reactor during [^{18}F]-fluoroethyl triflate production). Total synthesis time 100min.

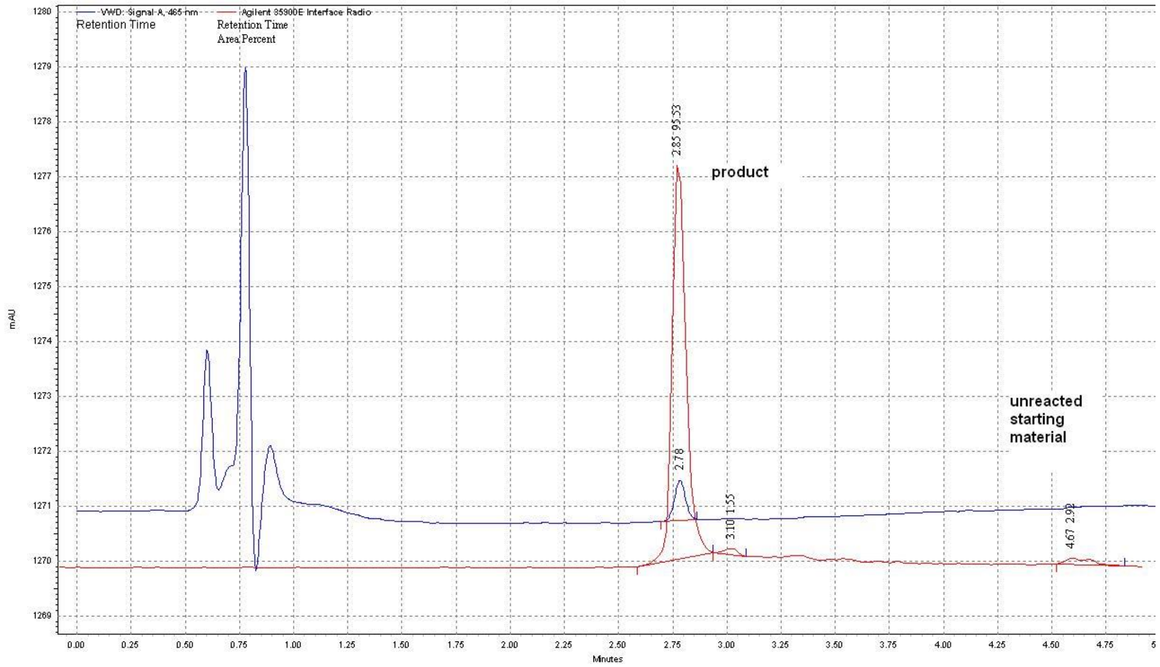

Figure S4. Typical HPLC chromatogram of the isolated [^{18}F]**3** after SPE purification coinjected with reference [^{19}F]**3**. Blue trace presents UV (465nm), red trace – radiodetector. Eluent ACN(containing 0.085% of TFA):TFA (0.1%) 10:90 gradient to ACN(containing 0.085% of TFA):TFA (0.1%) 90:10 in 4 min, then isocratic. Flow 2.5mL/min.

HPLC purification of [^{18}F]3.

The product solution could be injected on a preparative ACE 5 C18-HL (250x10mm) column, which was eluted at a flow 7.0mL/min with water for 2min, followed by ACN-0.1%TFA 37-63 mixture for 8 min and ACN-0.1%TFA 45-55 mixture. After 3 minutes with the latter mobile phase the product peak was collected. The product could be formulated in a solution suitable for injection as following: after dilution with water (50mL) the HPLC fraction was passed through an SPE cartridge (tC18, 1cc, 50mg). The cartridge was rinsed with H_2O (5mL) and eluted with EtOH (700µL) followed by PBS solution (7mL). RCY 13% (based on the total activity distilled to the reactor during [^{18}F]-fluoroethyl triflate production). Total synthesis time 130min.

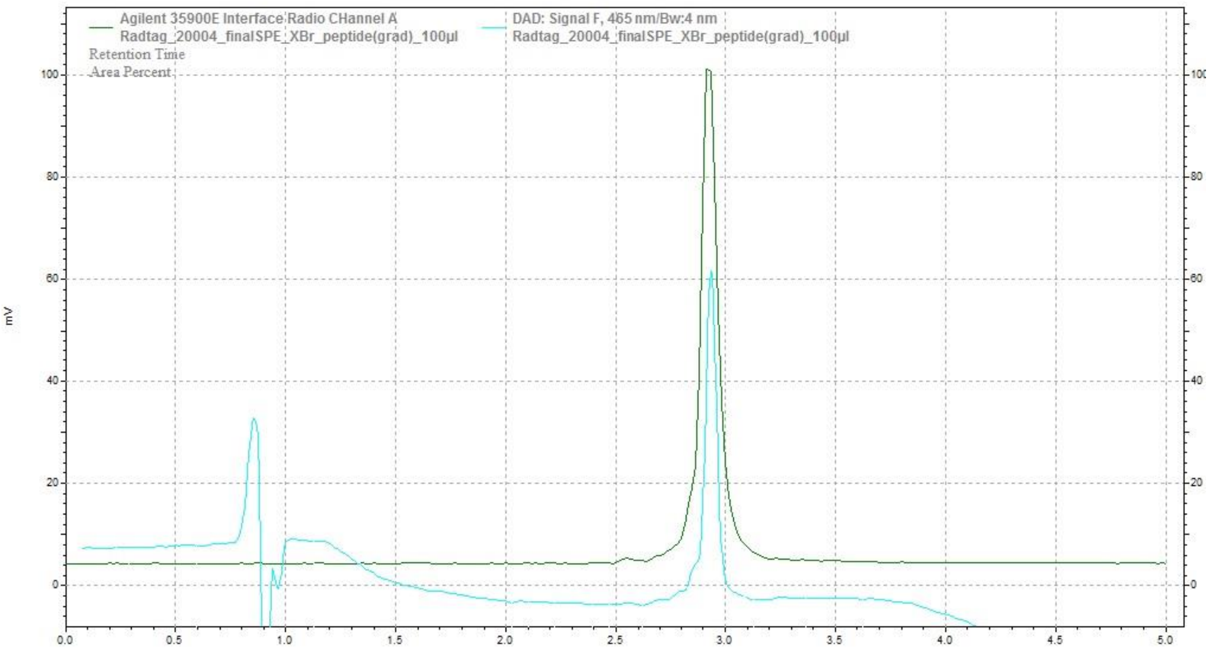

Figure S5. Typical HPLC chromatogram of the isolated [^{18}F]**3** after the HPLC purification coinjected with reference [^{19}F]**3**. Blue trace presents UV (465nm), green trace – radiodetector. Eluent ACN (containing 0.085% of TFA):TFA (0.1%) 10:90 gradient to ACN(containing 0.085% of TFA):TFA (0.1%) 90:10 in 4 min, then isocratic. Flow 2.5 mL/min.

PET-Imaging with mice

All mice were housed at the animal department of Karolinska University Hospital in a temperature (± 21°C) and humidity (± 40%) controlled environment on a 12h light/dark cycle (lights on 7:00 AM) with access to food and water ad libitum. Animals were allowed at least one week to habituate to the animal department before the start of the imaging sessions. All experiments were conducted during the light phase of the cycle. All experiments were performed in accordance with the guidelines of the Swedish National Board of Laboratory Animals under protocols approved by the Animal Ethics Review Board of Northern Stockholm, Sweden (N175/15).

Imaging Instrumentation

For the PET measurements, the Mediso nanoScan® PET-MRI and the nanoScan® PET-CT pre-clinical small animal imaging systems were used.[3,4] The two systems have identical PET performance[3] and were calibrated to provide consistent results. Two animals were measured at the same time in the two systems.

In vivo imaging

Male C57BL/6J mice have been injected subcutaneously with A375 or B16/F10 melanoma cells in the neck region. Ten days after inoculation the animals were anesthetized through inhalation of isoflurane (4-5% isoflurane in 100% oxygen). After induction the isoflurane concentration was lowered to 1.5-2% (50/50 air/oxygen) and the animals were positioned in the scanner in a designated mouse bed. A cannula was inserted in the tail vein through which the radioligand was administered. A 93-minute dynamic PET scan was initiated immediately upon intravenous injection of the radioligand.

Image- and Statistical Analysis

The acquired list mode data was reconstructed into 25 timeframes (93 min scan = 4x10 s, 4x20 s, 4x60 s, 7x180 s, 11x360 s). The image reconstruction was made with a fully 3-dimensional maximum-likelihood expectation maximization algorithm (MLEM) with 20 iterations, without scatter and attenuation correction. The reconstructed dynamic PET images were used to delineate VOIs for muscle and tumor regions in PMOD (PMOD Technologies Ltd., Zurich, Switzerland) to generate decay corrected time activity curves (TAC). The regional uptake values were expressed as standard uptake value (SUV), which normalizes for injected radioactivity and body weight.

PET imaging in a non-human primate

The study was approved by the Animal Ethics Committee of the Swedish Animal Welfare Agency (Dnr N452/11) and was performed according to the "Guidelines for planning, conducting and documenting experimental research" (Dnr 4820/06-600) at the Karolinska Institutet, the "Guide for the Care and Use of Laboratory Animals"), the AstraZeneca bioethics policy and the EU Directive 2010/63/EU.

A cynomolgus monkey (body weight 4.7kg) was used in the PET imaging. The NHP was housed in the Astrid Fagraeus Laboratory (AFL) of the Swedish Institute for Infectious Disease Control (AFL), Solna.

Anesthesia was induced by intramuscular injection of ketamine hydrochloride (approximately 10 mg/kg) at AFL and maintained by the administration of a mixture of isoflurane (1.5-2.0%), oxygen, and medical air with endotracheal intubation at the PET center.

PET measurement was conducted using a High-Resolution Research Tomograph (Siemens Molecular Imaging). The position of the NHP was arranged in order to put the body part as much as possible in the gantry.

A transmission scan of 6 minutes using a single ^{137}Cs source was performed before the [^{11}C]3 injection. List mode data were acquired continuously for 123 minutes immediately after intravenous injection of the radioligand. Images were reconstructed with a series of 34 frames (20 seconds × 3, 1 minute × 3, 3 minutes × 5, 6 minutes × 17) using the ordinary Poisson-3D-ordered subset expectation maximization (OP-3D-OSEM) algorithm.

Regions of interest (ROIs) were manually delineated on the liver, the lungs, the heart and the brain on the summation image. The time activity curves of each organ were generated by applying the ROIs to the dynamic PET data.

Binding assay data

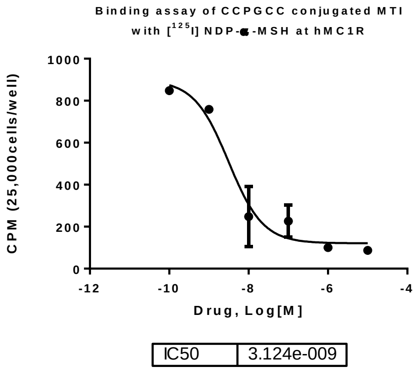

Figure S6. Competition binding assay of compound **3** with [^{125}I] NDP-α-MSH-peptide towards A375 melanoma cells.

Fluorescence staining data

Cell cultivation and the in vivo fluorescent measurement was done according to a previously published procedure.[5]

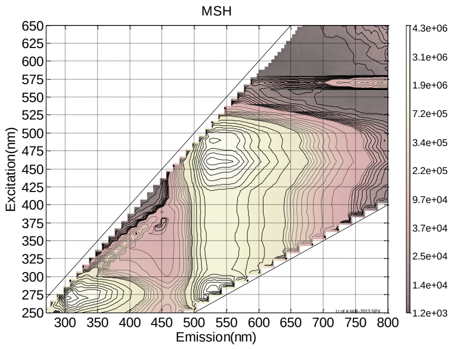

Figure S7. Excitation/emission spectrum of fluorescent probe **3**.

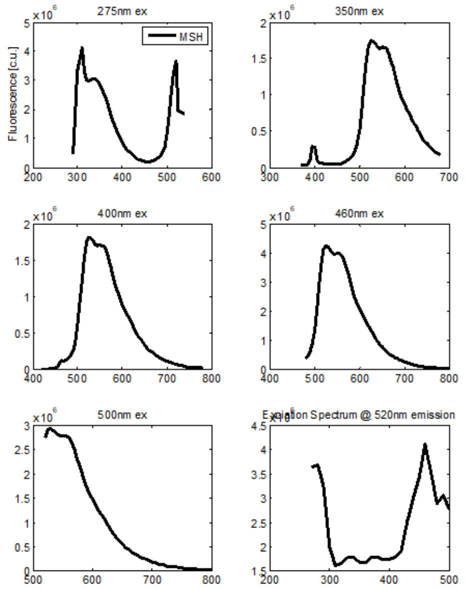

Figure S8. Various 2D- projections of the excitation/emission spectrum of fluorescent probe **3**.

References

1. S.R. Adams and R. Y. Tsien, "Preparation of the membrane-permeant biarsenicals FlAsH-EDT2 and ReAsH-EDT2 for fluorescent labeling of tetracysteine-tagged proteins." *Nat. Protoc.,* **2008**; *3(9)*: 1527-34.
2. M.-R. Zhang, K. Furutsuka, Y. Yoshida and K. Suzuki, "How to increase the reactivity of [^{18}F]fluoroethyl bromide: [^{18}F]fluoroethylation of amine, phenol and amide functional groups with [^{18}F]FEtBr, [^{18}F]FEtBr/NaI and [^{18}F]FEtOTf." *J. Label. Compd. Radiopharm.*, **2003**; *46*: 587-598. The RCY obtained by us according to this method in our laboratory were typically in range of 10-40% and synthesis time 35-40min.
3. K. Nagy, M. Tóth, P. Major, G. Patay, G. Egri, J. Häggkvist, A. Varrone, L. Farde, C. Halldin and B. Gulyás, "Performance evaluation of the small-animal nanoScan PET/MRI system." *J. Nucl. Med.,* **2013**, *54(10)*: 1825-32.
4. I. Szanda, J. Mackewn, G. Patay, P. Major, K. Sunassee, G. E. Mullen, G. Nemeth, Y. Haemisch, P. J. Blower, and P. K. Marsden, "National Electrical Manufacturers Association NU-4 performance evaluation of the PET component of the NanoPET/CT preclinical PET/CT scanner." *J. Nucl. Med.*, **2011**, *52(11)*: 1741-7.
5. M. Cai, Z. Liu, H. Qu, H. Fan, Z. Zheng, V. J. Hruby, "Utilize conjugated melanotropins for the earlier diagnosis and treatment of melanoma", *Eur. J. Pharm.*, 660 (1), **2011**, 188-93.
